# Treatment of Refractory Grover’s Disease With Dupilumab: A Case Study

**DOI:** 10.7759/cureus.88695

**Published:** 2025-07-24

**Authors:** Maclaine McCarter, David Cleaver, Jordan Lane

**Affiliations:** 1 Dermatology, A.T. Still University - Kirksville College of Osteopathic Medicine, Kirksville, USA; 2 Dermatology, Cleaver Dermatology, Kirksville, USA; 3 Dermatology, Northeast Regional Medical Center, Kirksville, USA; 4 Dermatology, Unity Health - White County Medical Center, Searcy, USA

**Keywords:** biologic therapy, dermatology case report, dupilumab, grover’s disease, interleukin-4 inhibition, refractory grover's disease, transient acantholytic dermatosis

## Abstract

Grover’s disease (GD) is a pruritic skin condition characterized by erythematous, scaly papules on the trunk, often resolving spontaneously but sometimes becoming persistent and difficult to manage. In this case study, we discuss a 73-year-old male with treatment-resistant GD who experienced significant improvement with dupilumab injections. Within two weeks, the patient reported noticeable improvement in pruritus and rash, with complete resolution achieved after five months of continued treatment and no notable adverse effects. This case can highlight dupilumab as a promising option for refractory GD, as well as support the hypothesis of an immune-mediated pathogenesis of GD.

## Introduction

Grover’s disease (GD), or transient acantholytic dermatosis, is a pruritic, papular, erythematous rash that most commonly affects the central chest and back. It was first described in 1970 by Ralph Grover, who had several patients with similar clinical and histopathological findings [1]. Although this is a benign skin condition, the pruritus can be intense and have a noticeable impact on the patient’s quality of life. Grover’s disease most commonly affects Caucasian males in the fifth decade of life or later, and histopathological examination classically shows acantholytic dyskeratosis. While its etiology remains unclear, Grover’s disease has been linked to other chronic dermatoses, such as atopic dermatitis, contact dermatitis, and xerosis cutis [2]. Grover’s disease should be differentiated from other skin disorders that are also histologically characterized by acantholysis such as Darier disease, Hailey-Hailey disease, and pemphigus.

Grover’s disease often resolves spontaneously within weeks to months, so management is primarily symptomatic relief. Although there are no randomized clinical trials of therapies for Grover’s disease, the most commonly-used options include topical corticosteroids, emollients, isotretinoin, acitretin, and systemic corticosteroids. Since heat and sweating may be triggers, patients with GD are advised to avoid activities that expose them to such triggers when possible [3]. Chronic treatment with some of these options, especially topical or systemic corticosteroids, can be concerning for long-term adverse effects, particularly in patients with multiple comorbidities. The association that GD has with atopic dermatitis has raised suspicion of a potential immune-mediated pathogenesis, and has consequently encouraged some clinicians to treat refractory GD with immune-modulators like dupilumab, an interleukin (IL)-4 and IL-13 inhibitor [4,5]. This manuscript herein presents the case of a 73-year-old patient diagnosed with Grover’s disease successfully treated with dupilumab injections.

## Case presentation

A 73-year-old male presented to the dermatology clinic with a three-week history of a rash affecting his central trunk. His previous medical history included depressive disorder, hypertension, history of transient ischemic attack (TIA), and history of basal cell carcinoma (BCC). The patient’s medication list included aspirin 81 mg, amlodipine 5 mg, and atorvastatin 80 mg, each taken once daily.

**Figure 1:**
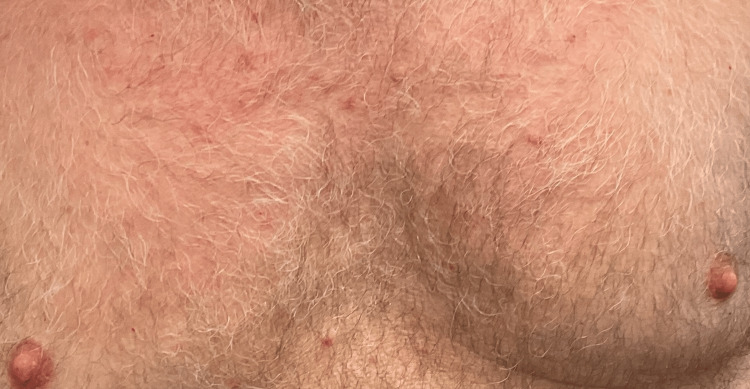
Erythematous papules scattered across the upper trunk prior to treatment initiation. Image courtesy of the treating dermatologist. Patient consent was obtained for publication.

The primary symptom he experienced was intense itching that was keeping him up at night. His primary care provider had originally prescribed him a 10-day regimen of doxycycline for what was originally thought to be folliculitis, but there was no improvement. A skin exam of the trunk revealed several erythematous, acuminate, scaly papules scattered across the upper back and chest, consistent with the clinical features of Grover’s disease (see Figure 1). After appropriate counseling, the patient was prescribed topical triamcinolone 0.1% cream to use twice per day, hydroxyzine 10 mg to use nightly, and encouraged to use over-the-counter antihistamines during the day as needed for the pruritus. 

**Figure 2:**
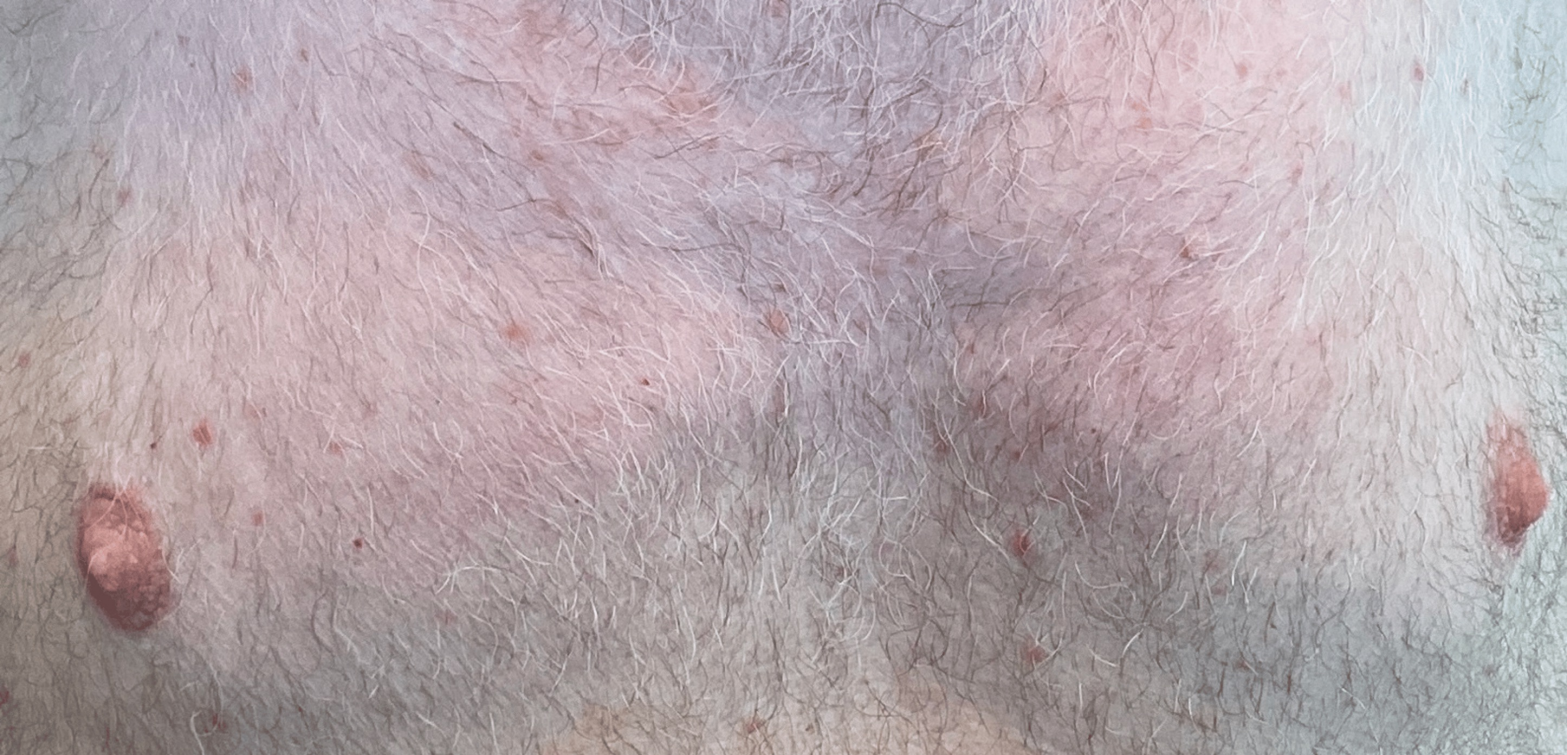
Resolution of the papular eruption following treatment with dupilumab. The precise interval between treatment initiation and this photograph is unknown. Image courtesy of the treating dermatologist. Patient consent was obtained for publication.

Three weeks later, the patient reported back to the dermatology clinic stating the rash had become worse and the triamcinolone cream provided no benefit. The patient was switched from triamcinolone to clobetasol 0.05% topical ointment, and a punch biopsy of the rash was performed. The dermatopathology report showed spongiotic dermatitis with focal acantholytic dyskeratosis, consistent with Grover’s disease. Due to the lack of improvement with topical corticosteroids, an Itch Numeric Rating Scale (NRS) of 10.0, and a total body surface area (BSA) of 20.0%, treatment with dupilumab was initiated. The patient received standard atopic dermatitis dupilumab dosing with a 600 mg subcutaneous injection on day 0, followed by 300 mg subcutaneous injections every 14 days thereafter. Just two weeks after the initial dose of dupilumab, the patient noted significant, but not complete, improvement of his rash, including relief of the pruritus. After five months of continuing his dupilumab injections, the patient reported to the clinic with well-controlled Grover’s disease that was no longer pruritic and denied any noticeable adverse effects to his injections. Four months later, at the next follow-up appointment, a skin exam revealed that his Grover’s disease had completely cleared. An image of the patient's trunk after initiation of dupilumab therapy is seen below in Figure 2.

## Discussion

This case highlights the potential ability to use an IL-4/13 inhibitor like dupilumab in the treatment of Grover’s disease that is refractory to the typical first-line therapies. While the exact cause of transient acantholytic dermatosis is unknown, the efficacy of dupilumab in its management can potentially provide some insight into the disease development, such as an immune-mediated pathogenesis. 

Several other case reports have described the successful use of dupilumab for refractory Grover’s disease. One report showed dupilumab’s efficacy in the treatment of three different patients, each with Grover’s disease that had failed several previous therapies, with resolution of their diseases within 12 weeks of starting dupilumab injections [4]. Similarly, another report detailed a 70-year-old female who failed several therapies for her acantholytic dermatosis, one of which was more than 20 treatments with narrowband UVB. After only six injections of dupilumab, she experienced complete resolution of her disease [5]. Our case adds to this growing body of evidence supporting dupilumab as a potential treatment for refractory Grover’s disease. 

Dupilumab is a humanized monoclonal antibody that binds to the alpha subunit of IL-4 receptors (IL-4Rα) and inhibits the downstream signaling of type 2 T lymphocytes (TH2), reducing the cytokine-induced release of proinflammatory cytokines, chemokines, nitric oxide, and IgE [6]. Due to its blockage of the cytokine-induced damage, dupilumab is commonly used to treat diseases that utilize the IL-4 and IL-13 pathway, including asthma, atopic dermatitis, chronic sinusitis with nasal polyps, and eosinophilic esophagitis. The effective treatment of Grover’s disease with dupilumab, as well as its association with atopic dermatitis, suggests a potential IL-4 cytokine-mediated pathogenesis. Additionally, there have been cases reported of patients experiencing Grover’s disease secondary to intravenous administration of IL-4 [7].

In addition to dupilumab, there are other treatments used for treating refractory Grover’s disease, such as systemic retinoids, systemic corticosteroids, and phototherapy [3]. Oral retinoids, including isotretinoin and acitretin, have been used successfully but carry an associated risk of increased mucocutaneous dryness, hepatotoxicity, and teratogenicity. One study evaluated four patients who were treated with oral isotretinoin for their Grover’s disease. Three of the patients responded positively with remissions up to 10 months after treatment, with one of them experiencing a relapse after the medication was stopped [8]. Systemic corticosteroids can provide relief, but are not ideal for long-term therapy due to their Cushing syndrome-related adverse effects, including weight gain, abdominal striae, and increased risk of osteoporosis and diabetes. Additionally, a systematic review found complete remission in only 64% of Grover’s disease patients who were treated with systemic corticosteroids [3]. Phototherapy is another alternative for refractory Grover’s disease, and is relatively safe. However, authors recommend the use of narrowband UVB therapy up to two to three times weekly until an appropriate response has been achieved [9], which may not be feasible for all patients due to cost.

## Conclusions

Grover’s disease, or transient acantholytic dermatosis, is a benign skin condition that has characteristic clinical and histopathological features, with potential of causing intense pruritus. This skin disease is associated with atopic dermatitis and has been shown to improve with the treatment of dupilumab, an inhibitor of the IL-4 and IL-13 immune pathway. While most cases are self-resolving with symptomatic treatment of topical corticosteroids and antihistamines, dupilumab may be a promising option for refractory Grover’s disease, though further studies are needed to confirm its efficacy. This case, as well as others, further support the notion that dupilumab can be a safe and effective treatment option in patients with refractory Grover's disease.
